# Relationship of growth differentiation factor-15 with aortic stiffness in essential hypertension

**DOI:** 10.2144/fsoa-2019-0029

**Published:** 2019-08-14

**Authors:** Erdoğan Sökmen, Cahit Uçar, Serkan Sivri, Mustafa Çelik, Kenan Güçlü

**Affiliations:** 1 Department of Cardiology, Kirsehir Ahi Evran University Training & Research Hospital, Kirsehir, Turkey; 2 Department of Internal Medicine, Kirsehir Ahi Evran University Training & Research Hospital, Kirsehir, Turkey; 3 Department of Biochemistry, Kirsehir Ahi Evran University Training & Research Hospital, Kirsehir, Turkey

**Keywords:** aortic elastic properties, echocardiography, GDF-15, hypertension

## Abstract

**Aim:**

We aimed to assess the relationship between echocardiographic parameters of aortic elasticity, namely aortic strain, aortic distensibility and aortic β-index, and serum growth differentiation factor (GDF)-15 in patients with newly diagnosed essential hypertension (HT).

**Methods:**

Grade-1 HT patients (n = 50), grade-2 HT (n = 70) patients and 35 healthy controls were included.

**Results:**

GDF-15 was greater in grade-2 HT group compared with the other groups. All aortic elasticity parameters were worse in grade-2 HT group compared with the other groups. GDF-15 correlated positively with E/E′ ratio (the ratio of transmitral E velocity to mean diastolic mitral annular velocity) and β-index; and aortic strain and aortic distensibility correlated negatively with serum GDF-15. β-index, aortic diastolic diameter and diastolic blood pressure were independently associated with GDF-15.

**Conclusion:**

GDF-15 may be utilized in the prediction of increased aortic stiffness.

## Lay abstract

Hypertension is a leading cause of morbidity and mortality worldwide, and is closely associated with impaired arterial and aortic elasticity. Growth differentiation factor (GDF)-15 is a novel marker of cardiovascular diseases, and appeals more and more to the researchers. In the present study, we evaluated the relationship of GDF-15 with echocardiographic parameters regarding aortic stiffness, namely aortic β-index, aortic distensibility and aortic strain in 120 newly diagnosed hypertensive patients. We found that serum GDF-15 was correlated with impaired aortic elasticity; moreover, it turned out to be a potent predictor of an increased aortic β-index in hypertension.

## Graphical abstract

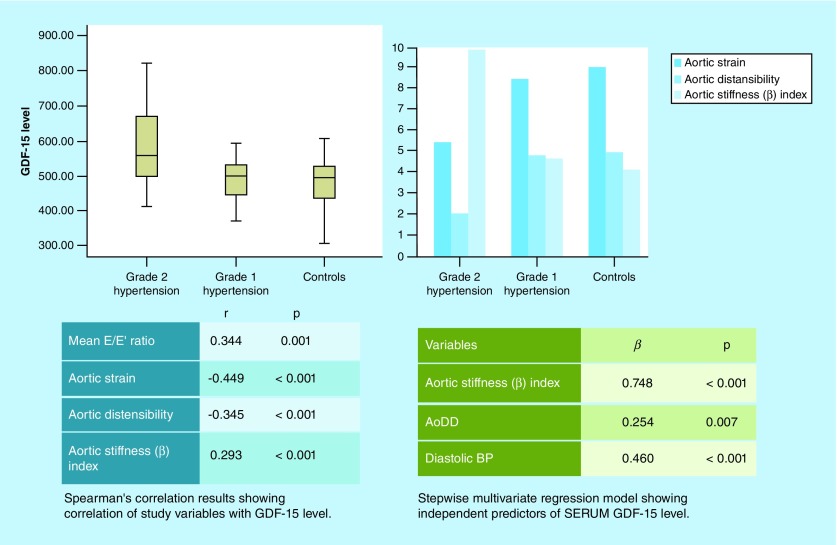

Hypertension (HT) is one of the leading causes of morbidity and mortality throughout the world [1]. Moreover, impaired aortic and arterial elasticity have proved to be a further contributor to the cardiovascular (CV) morbidity and mortality in subjects with HT [2], as well as in those subjects without other CV disease risk factors [3]. A close relationship between HT and impaired aortic elasticity has long been recognized, despite ongoing controversies as to whether aortic stiffness is the cause or the consequence of HT [4–6].

Growth differentiation factor (GDF)-15 is a member of the TGF-β cytokine superfamily [7], and recently it appeals more and more to the researchers worldwide owing to its prognostic and diagnostic significance in various disease conditions. While being secreted in a trace amount from a congeries of cells such as endothelial cells, vascular smooth muscle cells and macrophages under normal circumstances, diverse pathological conditions conducive to stress response may boost its secretion from both the cardiac and noncardiac tissues [8]. Although the exact underlying mechanism has yet to be totally known, a preponderance of evidence suggests elevated levels of GDF-15 in various disease conditions including acute coronary syndrome, stable coronary arterial disease, diabetes mellitus, solid cancers, essential HT, left ventricular hypertrophy in HT, atrial fibrillation, and acute and chronic heart failure [8–15]. Additionally, GDF-15 also emerged as an independent predictor of all-cause mortality in general population [16].

There are conflicting results with regard to the relationship between serum GDF-15 level and arterial stiffness and vascular dysfunction assessed by carotid–femoral pulse-wave velocity (CFPWV) [17], carotid-radial pulse-wave velocity and brachial flow-mediated vasodilation (FMD) [18,19] in community cohorts. However, the association of GDF-15 with aortic stiffness assessed by transthoracic echocardiography (TTE) has yet to be investigated. Therefore, we aimed in the present study to evaluate the relationship between GDF-15 level and aortic elastic properties via TTE in patients with newly diagnosed essential HT.

## Methods

### Study population

A total of 120 consecutive patients with newly diagnosed and untreated essential HT were prospectively enrolled in the study between May 2018 and December 2018. The patients were further divided into two subgroups as grade-1 HT (n = 50, mean age 48.9 ± 4.6 years) and grade-2 HT (n = 70, mean age 50.4 ± 5.3 years) groups. Healthy normotensive subjects (n = 35, mean age 48.5 ± 7.6 years) were included to constitute the control group. Demographic and clinical characteristics of the study population were obtained through physical examination, detailed history taking, echocardiography and laboratory analysis. BMI was calculated as weight in kilograms divided by the square of height in meters. The exclusion criteria were set as follows: reluctance to give informed consent, physical and clinical characteristics attributable to secondary HT, diabetes mellitus, severe kidney insufficiency, atherosclerotic CV disease, endocrine pathology, acute or chronic inflammatory diseases, alcohol use, being on anti-inflammatory or steroid therapy. Informed consent was obtained from each participant. Five patients with grade-3 newly diagnosed HT were also excluded from the study due to small number of the relevant group. Our study protocol complies with the ethical rules set by the Declaration of Helsinki, and the local ethics committee approved our study protocol.

Office blood pressure (BP) was measured from the brachial artery using a mercury sphygmomanometer (Erka Perfect Aneroid, Berlin, Germany) in a quiet environment, with the participant in a sitting position after at least 5-min rest. Additionally, two more measurements were performed with 5-min intervals to obtain a total of three BP readings. These readings were then averaged to end up with the ultimate BP measurement. All the measurements were performed in the morning between 8 and 11 am. The patients with averaged pressure readings ≥140/90 mmHg in the absence of any secondary disease condition likely to induce hypertensive response were diagnosed with essential HT [20]. The patients were subdivided into grade-1 and -2 HT groups, according to the mean pressure measurements (systolic pressure between 140 and 159 mmHg, and diastolic pressure between 90 and 99 mmHg for grade-1 HT; systolic pressure between 160 and 179 mmHg, and diastolic pressure between 100 and 109 mmHg for grade-2 HT).

### TTE & determination of aortic elastic properties

Echocardiographic assessment of the enrolled patients was implemented by using Vivid Echocardiography Device (Vivid S5, GE Vingmed Ultrasound AS, Horten, Norway). With the patient on the lateral decubitus position, parasternal long-axis imaging of heart was achieved, where dimensions and wall thicknesses of the ventricles, together with the diameter of left atrium, were measured, as per the relevant guideline [20]. Modified Simpson’s method was utilized to determine the left ventricular ejection fraction. Early (E) and late (A) transmitral inflow velocities and E-deceleration time (EDT) were measured from the apical four chamber view using pulse-wave Doppler imaging. In tissue Doppler imaging mode, a sample pulse-wave Doppler was placed on the lateral and septal mitral annular ends to measure respective early/late diastolic annulus velocities (E′ and A′ velocities, respectively). Transmitral E velocity was divided by the respective early diastolic mitral annulus velocities and hence septal and lateral E/E′ ratios (the ratio of transmitral E velocity to mean diastolic mitral annular velocity) were obtained. Echocardiographic examination for conventional measures was performed by complying with the standards by the American Society of Echocardiography [21].

Diameters of ascending aorta during systole and diastole were measured at a 3-cm distance from the aortic valve in M-mode from the parasternal long-axis view [22].

Systolic aortic diameter (AoSD) was measured when a maximal anterior aortic motion was observed, while diastolic aortic diameter (AoDD) was measured at peak of the electrocardiographic QRS complex (electrocardiographic deflection representing depolarization (activation) of the ventricles) [23]. Change in the aortic diameter was calculated by abstracting AoDD from AoSD.

The parameters indicating aortic elastic properties were calculated by taking the arithmetic mean of the relevant parameter recorded during at least three cardiac cycles. The following three parameters pertaining to the elastic property of aorta were defined as follows:

- Aortic strain (%) = (AoSD - AoDD) × 100/AoDD;

- Aortic stiffness (β) index = ln (SBP)/(DBP)/(AoSD - AoDD)/AoDD;

- Aortic distensibility (cm^2^/dyn^-1^.10^-6^) = 2 × (AoSD - AoDD)/AoDD)/SBP - DBP [24,25].

SBP: Systolic blood pressure; DBP: Diastolic blood pressure; Dyn: The abbreviation for dyne, a unit of force in the centimetre-gram-second system of physical units.

### Laboratory analysis & measurement of GDF-15

After at least 12 h of hunger, blood samples were drawn through venipuncture into gel-containing serum tubes between 8:00 am and 10:00 am, and the serum was separated from the cells by centrifuging at 1500 × *g* for 10 min. The serum was stored at -80°C until assayed. Before analysis, the samples were thawed at room temperature. Serum GDF-15 levels were measured by using an ELISA via a commercially available assay (www.relassay.com, Gaziantep, Turkey) in accordance with the manufacturer’s instructions. LOD of the assay was 5.09 ng/l. The intra- and interassay CVs for GDF-15 were <10 and <12%, respectively.

Hematological parameters were determined by using Sysmex XN-1000-automated blood cell counter (Sysmex XN-1000, Sysmex Corporation, Kobe, Japan). Routine serum biochemical parameters were measured using standard laboratory methods via Roche Cobas 8000 Autoanalyser (Roche Diagnostics, Mannheim, Germany).

### Statistical analysis

The statistical analysis was performed using SPSS (Version 21.0. IBM Corp., NY, USA). Quantitative data were assessed for normality by using Kolmogorov–Smirnov and Shapiro–Wilk tests. Continuous variables were expressed as mean ± standard deviation and median (25–75 Interquartile range, IQR), and categorical variables were expressed as numbers (percentages). The groups were compared by using Chi-square test, Kruskal-Wallis H test or one-way analysis of variance (ANOVA), on the basis of the type of the variable intended to be compared. Comparison of the groups between which statistical difference was observed via analysis of variance was made by use of Duncan multiple comparison test. Moreover, pair-wise comparison of the groups between which Kruskal–Wallis H test revealed statistically significant difference was performed using Mann–Whitney U test, and the findings were assessed after adjustment with the Bonferroni correction. The relationship between GDF-15 and the other variables in the study population was analyzed through Spearman’s correlation analysis. Finally, multivariate linear regression analysis was implemented so as to obtain the best predictive model for the variable of GDF-15. A two-sided p < 0.05 was accepted to indicate statistical significance.

## Results

A total of 120 consecutive patients diagnosed newly with HT were included in our study, of whom 70 (mean age 50.4 ± 5.3 years) were diagnosed as grade-2 HT and 50 (mean age 48.9 ± 4.6 years) were diagnosed as grade-1 HT. Moreover, 35 healthy normotensive subjects (mean age 48.5 ± 7.6 years) comprised the control group. Baseline clinical and demographic findings of the three groups are shown in Tables 1 and 2. As evident in the Table 1, there was no significant difference between the groups with regard to age, gender and BMI (p > 0.05). As for the laboratory and echocardiographic characteristics, there was no statistically significant difference between the groups regarding blood glucose level, alanine transaminase level, glomerular filtration rate and lipid parameters. In the comparison of the parameters of complete blood count, a significant difference was found between the groups with the lowest white blood cell counts and hemoglobin levels in the grade-2 HT group compared with the grade-1 HT and the control groups. Beyond this, platelet count, neutrophil count and red-cell distribution width were similar between the groups (p > 0.05). Serum GDF-15 levels were found to be significantly greater in grade-2 HT group compared with grade-1 HT and the control groups (569.5 ± 86 ng/l, 504.7 ± 52 ng/l and 501.4 ± 66 ng/l, respectively, p < 0.001). In pair-wise comparison, however, GDF-15 levels were similar between grade-1 HT and the control groups.

**Table 1.** Demographic and clinical characteristics of the study groups.

Characteristics	Grade-2 HT (n = 70)	Grade-1 HT (n = 50)	Healthy controls (n = 35)	p-value
Gender (female, %)	31 (44.2)	23 (46)	16 (45.7)	0.657
Age (year)	50.4 ± 5.3	48.9 ± 4.6	48.5 ± 7.6	0.200
BMI (kg/m^2^)	26.08 ± 1.73	25.68 ± 1.36	25.57 ± 1.63	0.228
Smoking (%)	20 (28.5)	14 (28)	11 (31.4)	0.712
GDF-15 (ng/l)^†^	569.5 ± 86a	504.7 ± 52b	501.4 ± 66b	**<0.001**
Glucose (mg/dl)	85.75 ± 4.10	85.71 ± 5.1	85.4 ± 7.2	0.897
GFR (ml/min/1.73 m^2^)	95.2 ± 14.8	96.5 ± 15.1	94.4 ± 15.6	0.722
ALT (U/l)	22.8 ± 12.9	24.4 ± 11.2	22.74 ± 8.7	0.262
Triglyceride (mg/dl)	162.78 ± 73.15	176.88 ± 77.14	173.48 ± 65.75	0.541
Total cholesterol (mg/dl)	193.7 ± 36.9	198.8 ± 30.7	198.2 ± 31.7	0.512
LDL-C (mg/dl)	108.5 ± 28.8	103.3 ± 27.3	112.6 ± 25.9	0.198
HDL-C (mg/dl)	44.7 ± 11.2	47.9 ± 11.9	44.3 ± 7.2	0.081
WBC (×10^9^/l)	7.77 ± 1.67a	8.35 ± 1.91ab	8.81 ± 1.64b	**0.030**
Hb (gr/dl)	14.54 ± 1.73a	15.27 ± 2.03ab	15.71 ± 2.21b	**0.022**
Plt (×10^9^/l)	285.40 ± 70.44	294.08 ± 68.98	296.22 ± 91.97	0.761
Neutrophil (×10^9^/l)	4.86 ± 1.82	5.14 ± 1.80	5.37 ± 1.53	0.420
RDW (%)	13.89 ± 2.46	13.56 ± 1.62	13.77 ± 1.68	0.676

† There is no statistically significant difference between the pairs marked with the same letter within the same line (p > 0.05).

P-values which are statistically significant are given in bold; The letters a, b and ab serve as the indicators by implying that pair-wise comparison of the groups with different letters reveal no statistical significance.

GDF-15: Growth differentiation factor 15; GFR: Glomerular filtration rate; Hb: Hemoglobin; HDL-C: High-density lipoprotein cholesterol; HT: Hypertension; LDL-C: Low-density lipoprotein cholesterol; Plt: Platelet count; RDW: Red cell distribution width; WBC: White blood cell count.

**Table 2.** Baseline echocardiographic features of the study groups.

Characteristics	Grade-2 HT (n = 70)	Grade-1 HT (n = 50)	Healthy controls (n = 35)	p-value
IVS (mm)	10.9 (10.2∼12.4)a	10.20 (9.8∼11.5)b	9.8 (8.9∼10.7)c	**0.022**
PWT (mm)	10.00 (8.90∼10.27)	9.30 (9.0∼10.0)	9.30 (8.90∼9.77)	0.394
LVEDD (mm)	46.2 (45.2∼50.2)	46 (45.1∼50.1)	45.7 (45.10∼50.2)	0.531
LVEF (%)	64.50 (61.3∼65.4)	64.0 (61.9∼65.1)	63.0 (65.0∼65.4)	0.574
LA (mm)	37.46 (35∼41.3)	34 (32.0∼37.9)	32.50 (32.0∼36.4)	0.179
Transmitral E velocity (cm/s)	65.3 (60.9∼78.8)	69.5 (47.1∼85.0)	67.90 (59.0∼83.40)	0.382
Transmitral A velocity (cm/s)	84.25 (70.1∼93.5)a	69.7 (50.3∼84.7)b	52.1 (48.00∼67.2)c	**<0.001**
E/A ratio	0.83 (0.70∼1.05)a	1.14 (0.81∼1.34)ab	1.24 (1.15∼1.37)b	**<0.001**
EDT (ms)	201 (165∼253)	192.00 (167∼236)	178.00 (168.50∼193.00)	0.073
Mean E/E′ ratio	8.32 (7.89∼9.44)	7.79 (5.8∼10.1)	7.33 (6.74∼8.08)	0.054
AoSD (mm)	33.20 (31∼34.2)	31.80 (31.3∼34)	32.50 (31.30∼34.00)	0.059
AoDD (mm)^†^	30.75 (29.2∼33.7)a	29.60 (28.4∼32.5)b	29.40 (28.40∼32.50)b	**0.019**
AoDC (mm)	1.85 (0.5∼2.3)a	2.20 (1.5∼2.9)ab	2.30 (1.50∼2.90)b	**0.002**
Systolic BP (mmHg)	147.3∼13.16a	135.8∼9.5b	119.61∼7.04c	**<0.001**
Diastolic BP (mmHg)	95.7∼7.7a	84.7∼8.2b	77.22∼6.97c	**<0.001**
Aortic strain (%)^†^	5.41 (3.13∼6.64)a	8.31 (7.32∼9.80)b	8.90 (7.52∼10.21)b	**<0.001**
Aortic distensibility (cm^2^. dyn^-1^. 10^-6^)^†^	2.02 (1.25∼2.72)a	4.78 (3.53∼5.89)b	4.90 (3.65∼6.90)b	**<0.001**
Aortic stiffness (β) index^†^	9.07 (6.37∼15.00)a	4.52 (3.78∼5.12)b	4.10 (3.70∼5.12)b	**<0.001**

† There is no statistically significant difference between the pairs marked with the same letter within the same line (p > 0.05).

P-values which are statistically significant are given in bold; The letters a, b, ab and c serve as the indicators by implying that pair-wise comparison of the groups with different letters reveal no statistical significance.

AoDC: Change in the aortic diameter; AoDD: Diastolic aortic diameter; AoSD: Systolic aortic diameter; BP: Blood pressure; dyn: The abbreviation for dyne; E/A: The ratio between the transmitral E and A velocities; EDT: E-deceleration time; E/E′: The ratio of transmitral E velocity to mean diastolic mitral annular velocity; HT: Hypertension; IVS: Interventricular septum; LA: Left atrium; LVEDD: Left ventricular end-diastolic diameter; LVEF: Left ventricular ejection fraction; PWT: Posterior wall thickness.

Mean systolic and diastolic office BPs of grade-2 HT, grade-1 HT and the control group were 147.3 ± 13.1/95.7 ± 7.7 mmHg, 135.8 ± 9.5/84.7 ± 8.2 mmHg and 119.6 ± 7/77.2 ± 6.97 mmHg, respectively (Table 2).

Echocardiographic findings of the three groups were presented in Table 2. Interventricular septal (IVS) thickness was significantly different between the three groups, with the highest IVS thickness in grade-2 HT group and lowest IVS thickness in the control group (p = 0.022). Posterior wall thickness, left ventricular ejection fraction, left atrium diameter, E velocity, E-deceleration time and mean E/E′ were similar between the groups. On the other hand, a pair-wise comparison of the three groups revealed significantly different mean A velocity and E/A ratio, with the highest mean A velocity and lowest E/A ratio in grade-2 HT group compared with the grade-1 HT and the control groups. Although AoSD was similar between the three groups, significantly greater AoDD and significantly smaller change in the aortic diameter were found in grade-2 HT group, compared with the grade-1 HT and the control groups. As expected, aortic stiffness (β) index was found to the greater in grade-2 HT group compared with the grade-1 HT and the control groups. Moreover, aortic strain and aortic distensibility were calculated to be significantly lower in the grade-2 HT and the control groups (p < 0.001). However, a pair-wise comparison of these three parameters pertaining to aortic elasticity, namely the aortic stiffness index, aortic strain and aortic distensibility, did not reveal any statistically significant difference between grade-1 HT and the control groups (p > 0.05).

### Correlation & regression analyses

According to the Spearman’s correlation analysis, serum GDF-15 level showed within the whole study population a significant and positive correlation with mean E/E′ ratio and aortic stiffness (β) index (r = 0.344, p = 0.001; and r = 0.293, p < 0.001; respectively). Furthermore, aortic strain and aortic distensibility were found to be significantly and negatively correlated with serum GDF-15 level (r = -0.449, p < 00.1; and r = -0.345, p < 0.001; respectively) (Table 3).

**Table 3.** Spearman’s correlation analysis demonstrating correlations between serum GDF-15 level and other variables.

Variables	Coefficient	p-value
IVS	0.111	0.206
PWT	0.090	0.304
LVEDD	-0.136	0.121
LVEF	0.146	0.094
LA	0.018	0.837
Transmitral E velocity	-0.073	0.423
Transmitral A velocity	0.127	0.160
E/A ratio	-0.074	0.416
EDT	-0.042	0.656
Mean E/E′ ratio	0.344	**0.001**
Aortic strain	-0.449	**<0.001**
Aortic distensibility	-0.345	**<0.001**
Aortic stiffness (β) index	0.293	**<0.001**

P-values which are statistically significant are given in bold.

E/A: The ratio between the transmitral E and A velocities; EDT: E-deceleration time; E/E′: The ratio of transmitral E velocity to mean diastolic mitral annular velocity; IVS: Interventricular septum; LA: Left atrium; LVEDD: Left ventricular end-diastolic diameter; LVEF: Left ventricular ejection fraction; PWT: Posterior wall thickness.

In stepwise multivariate linear regression analysis (*R* ^2^ = 0.82, p < 0.001), higher aortic stiffness (β) index, AoDD, and diastolic BP remained independently associated with greater serum GDF-15 level (Table 4).

**Table 4.** Results of stepwise multivariate linear regression model.

Variables	β	p-value
Aortic stiffness (β) index	0.748	**<0.001**
AoDD	0.254	**0.007**
Diastolic BP	0.460	**<0.001**

P-values which are statistically significant are given in bold.

AoDD: Diastolic aortic diameter; BP: Blood pressure.

## Discussion

Our study findings showed the following: GDF-15 level was significantly greater in patients with newly diagnosed grade-2 essential HT compared with those with grade-1 essential HT and healthy normotensive subjects; serum GDF-15 is positively correlated with aortic stiffness (β) index and mean E/E′ ratio, while negatively correlated with aortic strain and aortic distensibility; and increased serum GDF-15 was found to be an independent predictor for increased aortic stiffness (β) index, increased AoDD and increased diastolic BP.

The parameters of aortic stiffness and serum level of GDF-15 seem statistically similar between healthy controls and grade-1 HT patients. However, the same parameters and level were significantly different in grade-2 HT patients, as compared with the grade-1 HT patients and the control. At first glance, this situation may suggest a nonlinear distribution of the variables. However, arrhythmic means and medians only provide us with the point around which the statistical parameters gather, but not with whether there is a linear or nonlinear relationship between the variables.

Aortic stiffness shows a close association with such a number of conditions as atherosclerosis, acute coronary events, carotid intima-media thickness and end-stage renal disease [2,26–29]. There is a bidirectional association between aortic stiffness and HT [4–6]. General standpoint has been more inclined toward the hypothesis that aortic stiffness antedate the incident HT, and HT accelerates the deterioration of the aortic and arterial elasticity through increase in distension pressure, and promotion of matrix synthesis and hence vascular stiffening [30]. In favor of this hypothesis, some previous studies reported a deterioration in the aortic elasticity and increased aortic stiffness in normotensive subjects, which in turn led to increase in the prevalence of incident HT in the future [31,32]. Some other studies, on the other hand, reported increased aortic stiffness in patients with HT [33,34]. Since GDF-15 acts as a regulatory cytokine that assumes a CV protective role through activation of various cellular receptors and is implicated in inflammatory and apoptotic and fibrotic pathways [35–37], it will be prudent to assume increase in serum GDF-15 levels due to presence of endothelial dysfunction and inflammation in either situation.

Previous studies revealed conflicting results regarding the association of GDF-15 with arterial and aortic stiffness. In a study conducted on the Framingham Offspring cohort, CFPWV as a measure of aortic stiffness, augmentation index and forward pressure wave amplitude as a measure of stiffness in medium-sized arteries, and brachial FMD as a measure of endothelial dysfunction in medium-sized arteries were found to be significantly associated with an increased serum GDF-15 level [17]. Barma *et al*. [19], however, did not find any correlation of GDF-15 with arterial stiffness assessed by pulse-wave velocity and FMD. Another study by Lind *et al*. [18] also did not show any correlation between FMD and GDF-15 level; however, they revealed a significant association between GDF-15 level and carotid plaque burden, which is another predictor of CFPWV and hence aortic stiffness [38]. However, in the aforementioned studies, the mean ages of the patient population were older, namely between 61 and 76.8 years, compared with the mean age of around 50 years in our population. Moreover, their study populations included much more conventional CV risk factors compared with our study, which may also affect serum GDF-15 level and might have partially been responsible for the controversy in their findings of aortic and arterial stiffness. Beside this, we evaluated the aortic elastic properties by use of the three parameters measured by TTE. In this regard, our study is novel in terms of assessment of aortic stiffness by use of TTE in patients with newly diagnosed essential HT.

As for the association of GDF-15 and HT, there are only a small number of studies. Hanatani *et al*. [30] reported a significantly elevated serum GDF-15 level in patients with hypertensive left ventricular hypertrophy (LVH), compared with the healthy controls and the patients with hypertrophic cardiomyopathy. Similarly, Kou *et al*. [14] demonstrated in their recent study an elevated serum GDF-15 level in HT patients with LVH compared with the HT patients without LVH and healthy controls. However, GDF-15 level in their study was lower in HT patients without LVH compared with the healthy controls. In another study by Xue *et al*. [15], GDF-15 level in HT patients with LVH was found to be significantly greater compared with the HT patients without LVH. However, they did not include a control group in their study, in a similar manner to that of Kou *et al*. Although IVS thicknesses in our study were significantly different in a pair-wise comparison between the three groups with the highest IVS thickness in grade-2 HT group, this difference did not correlate with GDF-15 level in our study. Lack of correlation between IVS thickness and GDF-15 in our study may be explained by weakly significant p-value (p = 0.022) and similar posterior wall thickness between the three groups.

Greater burden of BP is associated with more impaired diastolic functions. In this regard, Dinh *et al*. [39] presented GDF-15 a novel biomarker of deteriorating LV diastolic functions, regardless of the presence of well-established risk factors related with Left ventricle (LV) diastolic dysfunction such as coronary arterial disease and HT. Positive correlation of mean E/E′ ratio with GDF-15 in our study is also compatible with the study by Dinh *et al.* [39].

The reason for the similarity between grade-1 HT patients and healthy normotensive subjects with regard to serum GDF-15 levels and the three echocardiographic indices of aortic elasticity may be attributable to the close proximity of BP readings of the grade-1 HT group to the diagnostic cut-off value for HT. Another explanation may be the possibility that a certain proportion of the patients in grade-1 HT group may actually possess white-coat HT. Since BP level is a well-known major determinant of aortic stiffness degree [40], it is quite likely to expect a prominent difference with regard to the three echocardiographic aortic stiffness parameters and serum GDF-15 level between grade-2 and -1 HT groups.

As mentioned previously, considering together the independent association of increased aortic stiffness with future CV events and the independent prognosis-predicting capability of GDF- 15 in diverse clinical conditions, it is rational to assume a close association between GDF-15 and impaired aortic elastic properties in patients with HT. Positive correlation of aortic stiffness (β) index and negative correlation of aortic strain and aortic distensibility with GDF-15, as well as a further independent association of aortic stiffness (β) index with GDF-15, in our study support this hypothesis. Moreover, this parameter is likely to be used as an indicator to recognize patients at higher risk of disease degree, aortic stiffness and carotid atherosclerotic burden. However, future studies with larger cohorts are warranted to confirm our hypothesis.

Our study should be interpreted with some limitations. Our study was performed in a single center and the population enrolled was relatively small. We did not relate our echocardiographic aortic elasticity indices with other parameters of vascular function and inflammation. We also relied solely on repeated office pressure measurements instead of an additional 24-h ambulatory BP monitoring, especially in the identification of patients with a probable white-coat HT.

## Conclusion

Our study showed that increased serum GDF-15 level was significantly correlated with increased aortic stiffness assessed by TTE. GDF-15 may prove to be a potent and significant predictor of aortic stiffness (β) index, diastolic BP and AoDD in patients with newly diagnosed HT. However, our study findings should be supported with future, large-scale studies.

## Future perspective

Although GDF-15 is a novel biomarker, studies concerning its relationship with various CV diseases are scanty and enrolled small patient populations. Furthermore, most of these studies were focused on GDF-15’s poor prognostic importance more than its possible diagnostic significance. On the other hand, new biomarkers for diagnostic purposes in CV disease conditions are needed especially in acute CV conditions. A normal reference range of serum level of GDF-15 is not well established. In this regard, we consider that further multicenter studies with larger patient cohorts should be conducted to define precise cut-off values for diagnostic, as well as prognostic significance of GDF-15.

**Summary points**

**Background & aim**

- Growth differentiation factor (GDF)-15, a member of the TGF-β cytokine superfamily, is a novel marker of cardiovascular diseases.
- Hypertension (HT) is one of the leading causes of morbidity and mortality throughout the world.
- Aortic strain, aortic distensibility and aortic stiffness (β) index are parameters assessed by transthoracic echocardiography and indicate the status of aortic elasticity.
- HT is linked to impaired aortic elasticity.
- Our aim was to assess the relationship between these aortic elasticity parameters and serum GDF-15 level in patients with newly diagnosed essential HT.

**Methods**

- One hundred and twenty consecutive HT patients and 35 healthy normotensive controls were included.
- HT patients were subdivided into grade-1 (n = 50) and grade-2 HT (n = 70) groups.
- Relevant data were gathered via physical examination, transthoracic echocardiography and laboratory analysis.
- GDF-15 was measured using ELISA.

**Results**

- Greater GDF-15 levels were found in the grade-2 HT group compared with the grade-1 HT and control groups (569.5 ± 86 ng/l, 504.7 ± 52 ng/l and 501.4 ± 66, respectively for GDF-15; p < 0.001).
- All three parameters were worse in grade-2 HT group compared with grade-1 HT and control groups.
- GDF-15 showed positive correlation with mean E/E′ ratio (the ratio of transmitral E velocity to mean diastolic mitral annular velocity) and β index; and, aortic strain and aortic distensibility were negatively correlated with serum GDF-15 level.
- In regression analysis, β-index, aortic diastolic diameter and diastolic blood pressure were independently associated with serum GDF-15 level (R^2^ = 0.82, p < 0.001).

**Discussion**

- Together with cardioprotective effect, GDF-15 can also serve as a poor prognostic indicator in various cardiovascular disease conditions.
- Data concerning diagnostic role of GDF-15 in cardiovascular disease conditions are not well known.

**Conclusion & future perspective**

- GDF-15 may be used in the prediction of increased aortic stiffness in HT patients.
- GDF-15 was a potent predictor of β-index, aortic diastolic diameter and diastolic blood pressure.
- Diagnostic importance of GDF-15 is not well established, and further multicenter studies with larger patient cohorts may help define the cut-off values for diagnostic and prognostic significance of GDF-15.
